# Leaf it to science: Uncovering plant immune systems through technological advances

**DOI:** 10.1093/plphys/kiaf615

**Published:** 2025-11-25

**Authors:** Xinnian Dong

**Affiliations:** Department of Biology, Duke University, PO Box 90338, Durham, NC 27708, United States; Howard Hughes Medical Institute, Duke University, Durham, NC 27708, United States

## Abstract

For the past 2 yr, I have been contemplating how best to write this review that not only reflects on a few significant and nostalgic moments in my more than 3 decades of professional career in the molecular plant–microbe interaction field, but also offers my personal outlook on the future aimed at inspiring young scientists to join this exciting discipline. Rather than a comprehensive overview, I would like to place greater emphasis on the “whys” and the “hows” than the “whats.” I finally decided to use technological advancements critical for the development of our field as a thread to connect the past with the future.

## My introduction to the molecular plant–microbe interaction field

Trained in microbiology (Dong et al. 1985, 1987, 1988, 1989; Womble et al. 1985), where the earliest molecular genetic tools were developed, my interest in plant–microbe interactions began with a fundamental question: Do plants have immunity? To me, the answer came in 1994 with the 4 publications on the cloning of the first resistance (*R*) genes in tobacco, tomato, and the model plant *Arabidopsis thaliana,* conferring resistance to specific effectors from tobacco mosaic virus (TMV), *Cladosporium fulvum*, and *Pseudomonas syringae*, respectively (Bent et al. 1994; Jones et al. 1994; Mindrinos et al. 1994; Whitham et al. 1994). Before then, single Mendelian dominant resistance loci had been used in agricultural breeding programs (Flor 1942, 1971). But I needed molecular proof to be convinced that plants possess intrinsic (innate) immune mechanisms. Following my postdoctoral work in Dr. Fred Ausubel's lab, where we were among the earliest to establish the *Arabidopsis–P. syringae* host–pathogen system (Dong et al. 1991; Whalen et al. 1991), I chose to focus on “basal resistance,” which is not pathogen- or pathogen effector-specific, in my own lab at Duke University. At the time, I was concerned that the so-called “gene-for-gene” resistance found in breeding programs (Flor 1971) might stem from polymorphisms in random genes, not representing common immune mechanisms. The cloning of the first *R* genes, which encoded conserved nucleotide-binding site and leucine-rich repeat domains, NB-LRRs, with either an N-terminal coiled-coil or a Toll/interleukin-1 receptor (TIR) domain, demonstrated that common innate immune mechanisms mediated by pathogen- or pathogen effector-specific receptors exist in plants. A few years later, similarly structured innate immune receptors were identified in *Drosophila* and mammals (Lemaitre et al. 1996; Poltorak et al. 1998), demonstrating that innate immunity is ubiquitously present in all multicellular organisms. The recent discovery of TIR domain-containing proteins in bacteria for antiphage defense indicates that similar innate immune mechanisms may have even deeper evolutionary roots (Wang et al. 2024).

With the effector-triggered immunity (ETI) shown to be mediated by NB-LRRs, an outstanding question that was still open at the time was: What is the molecular basis of basal resistance? To address this question, I constructed a transgenic *Arabidopsis* line carrying a pathogen-inducible reporter in which the transcription of β-glucuronidase (GUS) is driven by the promoter sequence of the antimicrobial gene, β-1,3-glucanase gene 2 (*BGL2*) (Keen and Yoshikawa 1983; Mauch et al. 1988a, 1988b; Mauch and Staehelin 1989). Based on the *BGL2:GUS* reporter expression pattern, I could more accurately quantify the resistance response using 4-methylumbelliferyl-β-D-glucuronide, which upon cleavage by GUS, releases a fluorescent product (Levvy and Marsh 1956; Jefferson et al. 1987). When I made the reporter line during my last year of postdoctoral training, I did not realize it would later become a major tool for launching my independent research career.

The fortuitous event (there were many more later) that facilitated this was the publication by Uknes et al. (Uknes et al. 1992) in which the authors showed that the *PATHOGENESIS-RELATED GENE* 2 (*PR2*) induced during systemic acquired resistance (SAR) (Ross 1961) was identical in sequence to the *BGL2* marker gene reported in my paper (Dong et al. 1991). Upon reading the SAR literature, I realized that this inducible broad-spectrum resistance was exactly what I was looking for as a topic for my own research project. There were so many intriguing questions that came to my mind: How does a local infection lead to systemic resistance? What is the mobile signal? What makes it broad-spectrum? How can we enhance SAR to control disease in crops? To address these questions, I already had a SAR-responsive reporter, *BGL2:GUS*, in the genetically trackable *Arabidopsis* plants. Even better, salicylic acid (SA) had been found to be the endogenous signal necessary and sufficient for inducing SAR (Malamy et al. 1990; Metraux et al. 1990; Gaffney et al. 1993). SA or its synthetic analogs, 2,6-dichloroisonicotinic acid and benzothiadiazole, could be exogenously applied to plants to activate SAR (White 1979; Ward et al. 1991; Friedrich et al. 1996; Gorlach et al. 1996; Lawton et al. 1996), instead of using a pathogen carrying complex signaling molecules, thus creating an ideal experimental system for performing reductionist research. The path forward became clear for me then.

## Genetic strategies for identifying key immune regulators in plants

It is not surprising that *R* genes were among the first cloned resistance-associated genes because they had been shown through crop breeding to be single-locus traits, ideal for application of classic genetic tools. The brilliant genetic selection strategy used to clone the tobacco *N* gene against TMV is my personal favorite (Whitham et al. 1994) (Table 1). Normally, *N* gene-mediated resistance would lead to localized programed cell death (PCD), also known as the hypersensitive response (HR), at the infection site. This response prevents the spread of the virus, limiting damage to local necrotic spots and keeping the rest of the plant healthy. However, incubation of infected plants at a higher temperature inactivates N, allowing the virus to spread systemically (Ross 1961). When such plants were moved back to the permissive temperature, restoration of wild-type (WT) N activity would lead to killing of the entire plant, whereas the N mutants that failed to mount the HR would survive, though with disease symptoms.

**Table 1.** Technologies described in the review.

Technology	Key findings	Method advantages	Limitations and considerations	References	PMID
Genetic selection	Identification of the N gene conferring resistance against TMV	Direct link to gene function	Pleiotropic effects on background; challenges with functional redundancy and essential genes	Whitham et al. (1994)	7923359
Forward genetic screen	Identification of the *npr1* mutants and other key immune genes (eg EDS1, SID2)	Direct link to gene function	More time consuming than genetic selection; pleiotropic effects on background; challenges with functional redundancy and essential genes. Consideration for proper mutant controls and rigorous statistical methodology	Cao et al. (1994) and Parker et al. (1996)	12244227; 8953768
Yeast 2-hybrid	Identification of TGA family of TFs as NPR1 interactors and RIN4 as targets of both pathogen effectors and host immune receptors	Identification of interactors through physical interactions in yeast	Requires retesting of the interaction in native organisms and subcellular compartments	Zhang et al. (1999) and Mackey et al. (2002)	10339621; 11955429
Reverse genetics	Identification of RPP4-mediated ETI-output genes	Hypothesis driven	Requires sound preliminary data on candidate genes and high-throughput functional analysis	Wang et al. (2011)	21293378
Microarray and RNA-seq	Identification of NPR1 direct transcriptional targets	Global profiling of transcriptomic changes	Costly and lower throughput in early studies	Wang et al. (2006)	15890886
RASL-seq	Time-course transcriptomic analysis of ETI-output transcripts	Direct quantification ∼500 mRNAs in ∼500 samples	Limited to the genes of interest	Wang et al. (2011) and Karapetyan et al. (2025)	17096590; 40928881
Quant-Seq	Time-course transcriptomic analysis mediated by NPR1 in response to SA	Inexpensive and relatively high throughput	Limited to only 3′ sequence of mRNAs	Powers et al. (2024)	39180213
RoGFP	Real-time detection of the redox rhythm and ETI-induced redox changes in different cellular compartments	Allows real-time live imaging of redox changes in different cellular compartments	Requires transformation of the sensor into the organisms	Karapetyan et al. (2025)	21293378; 40928881
Cryo-EM	Solved the structures of NPR1 and its complex with TGA3 TF	Eliminates the need for crystal formation	Facility required is not widely available	Kumar et al. (2022)	35545668
TurboID	Identification of components of the NPR1-enhanceosome	More sensitive than IP-MS in identifying transiently interacting proteins	Identified proteins may not be direct interactors of the probe protein	Powers et al. (2024)	39180213
GreenCUT&RUN	Identification of NPR1 binding sites in the genome	Much higher sensitivity than ChIP-seq	Requires GFP-tagged protein	Powers et al. (2024)	39180213
Ribo-seq	Profiling of global translatomic changes during PTI and ETI, which led to the identification of the R-motif and regulatory uAUGs/uORFs	Enables examination of translational activity on mRNAs	Low throughput and requires high sequencing depth due to rRNA contamination	Xu et al. (2017a), Yoo et al. (2020) and Xiang et al. (2023)	28514447; 31568832
SHAPE-MaP	Profiling of global RNA structural changes during PTI and identification of uAUG-ds	Allows examination of RNA secondary structural changes both in vivo and in vitro	A methodology under development that requires advanced data analysis	Xiang et al. (2023)	37674078

To identify SAR mutants (or to define genes causally related to any biological function), a genetic screen has to be employed. The biggest challenge with screening plant immunity mutants is that plant defense responses are significantly impacted by other factors, such as the physiological state of the plants (eg the age of the plant) (Kus et al. 2002), the level of the pathogen inoculant (Glazebrook et al. 1996) and the way how pathogen is applied (Zipfel et al. 2004), as well as the plant growth conditions, and other environmental factors (Zhu et al. 2010; Xin et al. 2016). To overcome these challenges, I chose to do my screen of the *Arabidopsis* mutants carrying the *BGL2:GUS* reporter on Petri dishes with or without SA. From this screen, we obtained mutants with constitutive *PR* gene expression without SA (*cpr*) and mutants that are nonresponsive to SA-induced *PR* gene expression (*npr*) (Bowling et al. 1994; Cao et al. 1994) (Table 1). Among the mutants, *npr1* has become a major focus for my lab's research as well as across the field, because it defines a step required for SA-mediated basal defense and SAR, and for its central role in the plant defense network (Panstruga et al. 2009; Backer et al. 2019; Zavaliev and Dong 2024). In parallel, a screen using the *PR1* reporter for SA-insensitivity has led to the identification of *sai1*, which is allelic to *npr1-1* (Shah et al. 1997). In addition to using reporters, screens using pathogens, such as *Hyaloperonospora arabidopsidis* (*Hpa*), which can be sprayed onto *Arabidopsis* seedlings after pretreatment with SA have several advantages, such as the ease of inoculation and relatively more homogenous plant morphology at the seedling stage. These have resulted in the identification of key immune gene mutants such as multiple alleles of non-inducible immunity (*nim1*), which turned out to be mutants of *NPR1* (Delaney et al. 1995).

Another screening strategy is the so-called enhanced disease susceptibility (*eds*) screen using low-dose pathogen inoculant to define mutants that are more susceptible than their already susceptible parents. This led to the identification of multiple key immune regulator genes, such as *EDS1* required for ETI and basal resistance, *EDS16/SID2* involved in SA synthesis (Glazebrook et al. 1996; Parker et al. 1996; Nawrath and Metraux 1999; Dewdney et al. 2000) as well as additional mutant alleles of *NPR1* (*npr1-2*, *npr1-3*, and *npr1-4*) (Cao et al. 1997) (Table 1).

**Figure 1.**
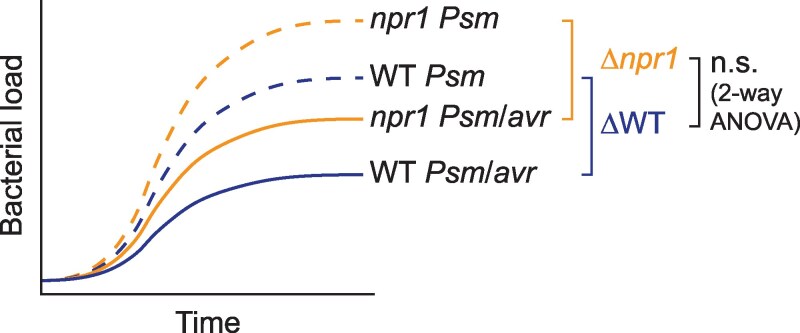
A diagram demonstrating the necessity of the 2-way ANOVA analysis to correct for the background differences between the WT and the *npr1* mutant for evaluating their response to immune induction. For example, WT and *npr1* plants are challenged by *P. syringae* pv. *maculicola* (*Psm*) with and without the effector (Avr). Even though, compared to the WT, higher bacterial growth is observed for both *Psm* and *Psm*/*Avr* in the *npr1* mutant due the deficiency in basal resistance, the difference in bacterial growth observed between *Psm* and *Psm/Avr* in the *npr1* mutant due to ETI is similar to that observed in the WT, indicating that the mutant is not deficient in ETI. n.s., not significant. This figure was generated by Y. Xiang.

While genetic approaches are favored by plant biologists because when they work, they are highly effective, one of the caveats of using genetics (more described below) is the pleiotropic physiological effects of a mutation over developmental time. For the molecular plant–microbe interaction field, the saving grace for this problem is our focus on the response to immune induction, which normally occurs within minutes to a few days. Therefore, the response of the mutant can be compared to itself as the background in the absence of induction, and the difference observed in the mutant can then be compared to that observed in WT using a 2-way ANOVA test, which allows for comparing the “differences of the differences” between the genotypes in response to immune induction. Otherwise, one could be led to completely wrong conclusions. For example, to determine whether NPR1 is required for ETI, examining the growth of a bacterial strain carrying an effector in the *npr1* mutant would have led to the conclusion that it is a positive regulator of ETI due to the significantly more bacterial growth compared to that observed in WT plants. However, if we compare the growth of the same bacterial strain with and without the effector in the *npr1* mutant, the difference in bacterial growth due to ETI would be similar to that observed in the WT, indicating that NPR1 is not required for ETI, and the upshifted pathogen growth in the mutant is due to the deficiency in basal resistance (Fig. 1). In fact, we and others found that NPR1 is a negative regulator of ETI (Rate and Greenberg 2001; Fu et al. 2012; Liu et al. 2016; Zavaliev et al. 2020). In addition to using the right controls and statistical analyses, another solution for reducing the pleiotropic effects of a mutation is to generate conditional knockdown lines, even though it may complicate the immune induction procedure.

## Molecular identification of functional partners through yeast 2-hybrid or south-western screens

Another limitation in using genetics is that it is less effective in discovering families of potentially redundant regulators, such as transcription factors (TFs), or cellular components whose mutations cause lethality. Molecular tools such as yeast 2-hybrid (Y2H) and South-Western screens have filled these gaps and led to the discovery of key TFs, such as TGAs, which are NPR1 interactors, and WRKYs, which bind to the *PR1* promoter sequence (Rushton et al. 1996; Zhang et al. 1999; Després et al. 2000; Zhou et al. 2000; Kim and Delaney 2002) (Table 1).

Y2H has also led to the identification of RPM1-INTERACTING PROTEIN 4 (RIN4) (lethal when mutated in the WT background) by Jeff Dangl's lab as a host target of the bacterial effectors AvrRpm1 and AvrRpt2, as well as their cognate host NB-LRR immune receptors, RPM1 and RPS2, respectively (Mackey et al. 2002 , 2003) (Table 1). Identification of RIN4 provided additional molecular evidence for the “guard hypothesis” developed earlier also using Y2H for AvrPto–Pto–Prf interaction as a model in which NB-LRRs serve as “guards” of host targets of pathogen effectors (Frederick et al. 1998; Oldroyd and Staskawicz 1998; Van der Biezen and Jones 1998; Dangl and Jones 2001). Y2H was then extended to generate a plant–pathogen protein–protein interactome network to prove the convergence of pathogen effectors onto host defense signaling hubs (Mukhtar et al. 2011). Together, the “guard” and the “decoy” hypotheses (van der Hoorn and Kamoun 2008; Zhou and Chai 2008; Zipfel and Rathjen 2008) explain how a limited number of *NB-LRR* genes in plant genomes can provide sufficient protection against an unlimited number of pathogen effectors by converging on recognizing those key host targets that are worth guarding or mimicking (as decoys).

## Using genomic approaches to gain a global view of plant immune responses

The dawn of the genomic era at the turn of the millennium has broadened our view from individual genes to the whole genome level, with the development of techniques for transcriptome profiling: microarray, RNA-seq, and other more high-throughput methods described below. To efficiently extract information from the large amount of “seq” data requires not only new bioinformatics analysis tools, but also careful experimental designs and pilot runs. Otherwise, the large dataset may become an expensive trap, instead of a roadmap for the project. Success is more likely to be achieved when reductionistic approaches are applied to the experimental strategy before the advent of the artificial intelligence era.

We used one such reductionistic approach to identify those SA-mediated genes whose expression is directly regulated by NPR1. To control NPR1 nuclear translocation, we constructed the NPR1-GR transgenic line in which the nuclear translocation of NPR1 is under the control of the glucocorticoid receptor (GR) hormone binding domain. Used in combination with the translation inhibitor, cycloheximide, we captured those genes whose transcription occurred without new protein production when NPR1 was allowed into the nucleus. We found that NPR1 directly regulates both the antimicrobial *PR* genes and the ER-resident genes (*ER* genes) required for proper protein modification and secretion (Wang et al. 2005) (Table 1). The significance of this coordinated regulation by NPR1 became more apparent when I attended “Stress Proteins in Growth, Development and Disease” Gordon Research Conference in 2005, organized mainly for yeast and animal biologists. There I learned that besides their role in unfolded protein response, these *ER* genes play a key physiological role in preventing the formation of unfolded proteins (Wang et al. 2005). This made me realize the intricate interplay between plant immune responses and overall plant physiology. With NPR1 inducing both *PR* and *ER* genes in response to SA, plants not only synthesize antimicrobial proteins to combat infection, but also ensure their safe deployment to the front lines to fight infection, while protecting themselves from collateral damage. My research has since been shifted from focusing only on understanding plant defense mechanisms to studying their interplays with other physiological processes, a direction that is currently gaining more momentum.

While earlier RNA-seq methods enabled global transcriptome profiling, the relative high costs limited their frequent use. This created a technology gap between reverse transcription quantitative polymerase chain reaction (RT-qPCR), which analyzes 1 transcript at a time, and RNA-seq, which captures the entire transcriptome. As the number of genes of interest and the number of samples increase, the cumulative cost of RT-qPCR can become prohibitively high. A good alternative is Quant-Seq, which sequences only the 3′ end of mRNA for RNA quantification (Moll et al. 2014). This cost-effective approach allows global transcriptome profiling in up to 300 samples in a single sequencing run (Table 1).

Another significant bottleneck in genomic analysis is the development of high-throughput reverse genetic tests of candidate genes, which are normally numbered in hundreds, if not thousands. Instead of cell cultures, which are not widely used in plant research, the transient expression systems in *Nicotiana benthamiana* (Goodin et al. 2015; Derevnina et al. 2019) and *Arabidopsis* protoplasts (He et al. 2007) have been very effective in testing gene functions in transcriptional and translational regulation, molecular interactions, plant immune responses, such as ETI-associated PCD, as well as studying metabolic pathways, such as those involved in SA synthesis (Liu et al. 2025; Wang et al. 2025; Zhu et al. 2025).

Functional genomic analyses also provided opportunities to identify immune-output genes that normally work in concert to confer pathogen resistance. Years of research have discovered many immune-associated cellular outputs. Besides ETI-associated HR, there are ROS (reactive oxygen species) accumulation (Torres et al. 2006), callose deposition, calcium influx, the production of antimicrobial PR proteins, and metabolites (known as phytoalexins) (Ahuja et al. 2012; Xu et al. 2022), to name a few. However, mutating individual genes often leads to a partial defense phenotype or no phenotype at all, making it difficult to confidently define the function of these genes in pathogen resistance. One strategy of reverse genetic analysis developed in our study (Wang et al. 2011) characterized 106 transcriptionally induced candidate gene mutants based on transcriptomic analysis of RPP4 (an NB-LRR)-mediated ETI against *Hpa*. The ease of the *Hpa* inoculation method allowed us to quickly screen through candidate gene mutants (more than 1 allelic mutant per gene for most of the genes) that identified mutants of 22 genes showing different degrees of susceptibility to *Hpa*. To dissect the multiple steps of RPP4-mediated immune response, we scored the occurrence (%) of 7 resistance and susceptibility phenotypes for each mutant. By performing unbiased clustering analyses using the numerated phenotypes of each mutant (ie 7 numbers), the 22 mutants were clearly separated into 2 major clusters, with Cluster 1 involving genes that trigger cell death as the predominant contributor (63.8% of the eigenvector composition) to RPP4-mediated resistance against *Hpa* (Table 1).

**Figure 2.**
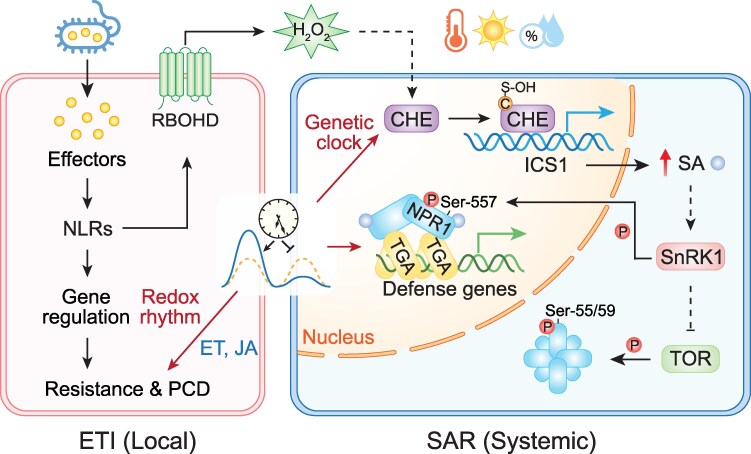
The interplay between plant immune responses and metabolic activities. ETI-associated PCD is gated by the redox rhythm with a stronger PCD observed in the subjective morning than in the subjective evening. This effect is regulated by the JA and ethylene (ET) signaling pathway. H_2_O_2_ generated during ETI through the activity of the membrane-associated NADPH oxidase RBOHD (respiratory burst oxidase homolog D) serves as a mobile signal to S-OH the circadian transcription factor CHE to enhance its binding to the promoter of *ICS1* to increase the synthesis of SA in systemic tissues. SA controls the downstream signaling component NPR1 activity through 2 master nutrient-sensing kinases, TOR and SnRK1. Under normal growth conditions, TOR keeps NPR1 inactive through phosphorylation at Ser55/59. During defense responses, elevated SA enhances the SnRK1 activity, which in turn inhibits TOR signaling and phosphorylates NPR1 at Ser557, which is necessary and partially sufficient for NPR1's nuclear function as a transcription cofactor for SA-mediated gene expression and resistance. This figure was generated by S. Karapetyan and Y. Xiang using BioRender.

I was particularly proud of this combined genomic and reverse genetic approach, which enabled the identification of 22 new genetically validated ETI-output genes in a single study, including ADR1-like 1, which has now been shown to be a helper NLR (Bonardi et al. 2011). However, reporting the functions of 22 genes turned out to be a much harder job than reporting on a single gene function; the manuscript had a difficult time getting published. To make the study more “attractive,” we performed promoter analysis of these ETI-output genes. To our surprise, the only enriched consensuses were “evening element” and “CCA1-binding site,” which are known binding sites for the core circadian clock TFs, CIRCADIAN CLOCK ASSOCIATED 1 (CCA1) and LATE ELONGATED HYPOCOTYL. This discovery threw the project, initially considered “complete,” into a new direction with several immediate questions: (i) Are these ETI-output genes regulated by the circadian clock? (ii) If the answer is yes, is there an interplay between RPP4-associated and the circadian clock-controlled transcription? (iii) What is the significance of circadian clock regulating these ETI-output genes? To answer these questions, we needed to re-analyze the transcriptional changes of all 22 ETI-output genes in the WT, *rpp4*, and *cca1* backgrounds with and without infection by *Hpa* with more frequent sampling in a much longer time course, ie 2-h sampling interval for 46 h. The large number of samples became another technical obstacle for this project. We overcame it by using an innovative high-throughput method: RNA annealing selection ligation-sequencing (RASL-seq) (Li et al. 2012), which allows quantifying expression of several hundred selected genes directly using mRNA extracts from hundreds of samples (Table 1). The RASL-seq data led to another surprising discovery: in the absence of *Hpa* challenge, the ETI-output genes are rhythmically expressed with the peak time at dawn and *Hpa* challenge led to more prolonged expression of these genes, indicating independent regulation by the circadian clock and the ETI-induction. These findings addressed both Questions (i) and (ii). To address Question (iii), we hypothesized that the ETI-output genes are pulse-expressed at dawn by the circadian clock to help plants anticipate peak infection risk because *Hpa*, an obligate biotrophic oomycete pathogen, disseminates spores only in the morning (Slusarenko and Schlaich 2003). Supporting this hypothesis, we found that plants had higher levels of resistance to *Hpa* if inoculation occurred at dawn than at dusk (Fig. 2).

Besides *Hpa*, plant interactions with other pathogens and insects as well as the basal synthesis of the defense hormones SA and jasmonic acid (JA) were also found to be impacted by the circadian clock (Roden and Ingle 2009; Bhardwaj et al. 2011; Goodspeed et al. 2012, 2013; Hevia et al. 2015; Zheng et al. 2015; Lu et al. 2017). In addition to anticipating infection, the circadian clock has been shown to gate or modulate the strength of immune responses depending on the time-of-day the pathogen challenge occurs (Bhardwaj et al. 2011; Korneli et al. 2014; Zhou et al. 2015; Karapetyan et al. 2025). Moreover, pathogen-induced SA synthesis in systemic tissue has been shown to be directly controlled by the circadian clock TF, CCA1 HIKING EXPEDITION (CHE) (Zheng et al. 2015; Cao et al. 2024). Although this activity of CHE is not directly mediated by its role in the circadian clock, but rather triggered by H_2_O_2_, which acts as a systemic signal to sulfenylate (S-OH) the protein and enhance its promotor binding (Cao et al. 2024). The involvement of a clock TF that moonlights in controlling SAR underscores, once again, the importance of timing in plant immune responses (Fig. 2).

## Linking immune responses to cellular metabolism through redox profiling

The intricate interactions between plant immune responses and the circadian clock indicate that plant defense is an integral part of overall plant physiology rather than a separate or supplementary process. To fully understand plant immune mechanisms, a holistic approach is essential. Shortly after our discovery of the clock's regulation of ETI-output genes, studies emerged reporting the existence of a circadian redox rhythm across all lineages of life, measured by the oxidation status of the antioxidant enzymes, peroxiredoxins (O'Neill et al. 2011; O'Neill and Reddy 2011; Edgar et al. 2012). These reports immediately piqued our interest, as we had previously found that the nuclear translocation of NPR1 is redox-regulated in response to SA-triggered perturbations (Mou et al. 2003; Tada et al. 2008). However, it has been difficult to dissect the distinct function of the redox oscillation because it is intricately intertwined with the genetic clock. For example, the transcription of *GLUTATHIONE SYNTHASE 1* encoding the enzyme involved in the synthesis of the major redox molecule, glutathione, is regulated by the circadian clock (Karapetyan et al. 2025). In the opposite direction, we found that perturbation of the redox rhythm by SA reinforces the genetic clock through NPR1 (Zhou et al. 2015). Although oscillations of molecules, such as peroxiredoxins and NADPH, were observed in the genetic clock-defective backgrounds, concerns remained about the rhythm being driven by unknown external factors (Abruzzi et al. 2021). Additionally, the lack of a clear physiological output regulated by the circadian redox rhythm has hindered the understanding of its biological significance. To disentangle the redox rhythm from the genetic clock, we performed concurrent metabolic and transcriptional time-course measurements in an *Arabidopsis* long-period clock mutant to detect coexistence of the 2 rhythms differentiated by their period lengths (Karapetyan et al. 2025). Besides the conventional measurement of oxidized and reduced glutathione ratio as a readout of the cellular redox status, which is a labor-intensive task, we also adapted the glutaredoxin-fused redox-sensitive GFP (roGFP2) reporters targeted to chloroplasts (Ugalde et al. 2021) to allow real-time, *in planta* monitoring of glutathione redox potential from different organelles (Table 1). Moreover, from the concurrent RASL-seq time-course data, we identified genes coordinately regulated by the redox rhythm based on their short period (∼24 h) in the long-period clock mutant (∼32 h) and showed that they were involved in gating of ETI-associated PCD toward the morning. This result indicated that the level of ETI-triggered cell death is time-of-day dependent, based on the cellular redox rhythm. Furthermore, we demonstrated that the signaling pathway responsible for this regulation involves the defense hormones JA and ethylene as well as redox changes in energy producing organelles tracked by roGFP reporters. Through this study, we propose that compared to robust genetic clocks, the more sensitive circadian redox rhythm coexists to serve as a signaling hub in regulating incidental energy-intensive processes, such as immune-induced PCD, which involves reprograming of chloroplast and mitochondrial activities (Karapetyan et al. 2025), to provide organisms with a flexible strategy to mitigate metabolic overload during stress responses (Fig. 2).

Though not involved in redox rhythm-mediated gating of PCD, SA does influence the redox and primary metabolism through various mechanisms. SA has previously been shown to affect plant metabolism through alternative respiration during thermogenesis via alternative oxidase (Raskin et al. 1989; Rhoads and McIntosh 1992), which contributes to plant stress resilience by maintaining redox and metabolic homeostasis (Vishwakarma et al. 2015). SA can also bind to CATALASE 2 to inhibit its scavenging activity on H_2_O_2_ (Chen et al. 1993), while the resulting redox changes induce the nuclear translocation of NPR1 (Mou et al. 2003). However, how metabolic pathways influence the SA-mediated immune response remained a significant knowledge gap. The answer to this question came unexpectedly through our search for kinases involved in NPR1 activation upon SA induction. When NPR1 was first cloned, the only sequence homology we could identify was with ankyrin repeats (Gorina and Pavletich 1996; Cao et al. 1997; Ryals et al. 1997). Aligning the NPR1 sequence with other ankyrin repeat-containing immune regulators in mammals, NF-κB precursor p105 and the inhibitor I-κB, led to the identification of conserved “I-κB degrons” in NPR1, with potential phosphorylation targets Ser11/15 and Ser55/59. We found that phosphorylation of Ser11/15 and Ser55/59 positively and negatively regulates the protein activity, respectively (Spoel et al. 2009; Saleh et al. 2015). Though the kinases that phosphorylate I-κB were identified in the same year that NPR1 was cloned (Mercurio et al. 1997; Régnier et al. 1997; Woronicz et al. 1997; Zandi et al. 1997), we only identified the kinases that control NPR1 activity 28 yr later in a comprehensive study of SA-induced NPR1 phosphorylation. The effort has led to the unexpected, but very exciting discovery: SA controls NPR1 activity through 2 master nutrient-sensing kinases, TARGET OF RAPAMYCIN (TOR) and SNF1-RELATED KINASE 1 (SnRK1) (Chen et al. 2025). Under normal growth conditions, TOR keeps NPR1 inactive through phosphorylation at Ser55/59. During defense responses, elevated SA enhances the SnRK1 activity, which in turn inhibits TOR signaling and phosphorylates NPR1 at Ser557. Ser557 phosphorylation is necessary and partially sufficient for NPR1's nuclear function as a transcription cofactor for SA-mediated gene expression and resistance (Fig. 2).

The integral role of SA in controlling central metabolic regulators SnRK1 and TOR to coordinate immune responses and growth through antagonistic modifications of NPR1 underscores the importance of such regulation to plant health, reminiscent of the role of NF-κB/IκB in human health (Zhang et al. 2017). It also raises the question: what is the metabolic cost of SA-induced immunity and what would happen if SA-mediated resistance becomes misaligned with cellular metabolic activities? The answer to this question came from our unexpected observation that keep SA-treated plants in prolonged darkness, when the plant is metabolically “starved” due to the lack of photosynthesis, led to massive loss of plant fresh weight and, ultimately, to plant death, while the control plants without SA treatment stayed alive under the same conditions (Zhou et al. 2015). At the 2023 Banbury Conference on the Future of Plant-Environment Interactions, I shared this observation with Joanne Chory. She immediately suggested pursuing a genetic selection for surviving mutants, to which I responded that we had already obtained such mutants. It felt like a “back to the future” moment, reminding me that Joanne, I, and many others in the field, had launched our careers using such classic genetic approaches. From this comprehensive genetic selection, we identified a large collection of survival of SA-induced death (*ssd*) mutants. Studying these mutants will help us uncover the deleterious effects of SA treatment and the mechanisms by which they are mitigated when the plants are grown under light/dark conditions.

These studies have provided a global perspective on how defense responses are monitored in plants. While a similar set of defense genes is activated upon pathogen challenge, the outcome of the responses is determined not only by the magnitude and duration of the immune induction (Yuan et al. 2021), but also by the context of the plant's metabolic state. Since plant metabolic activities exhibit daily oscillations (see review by Joanne Chory's lab; Seluzicki et al. 2017), fluctuations in primary and secondary metabolite levels, ROS production, and energy availability can profoundly impact the outcome of defense responses, sometimes determining survival vs. death (Zhou et al. 2015). Therefore, the direct regulation of immune signal synthesis and output genes by the circadian clock and the redox rhythm allows plants to optimize resource allocation and maximize plant health by avoiding conflicts between growth and defense (Fig. 2).

## Getting to the protein level using structural and proteomic approaches

**Figure 3.**
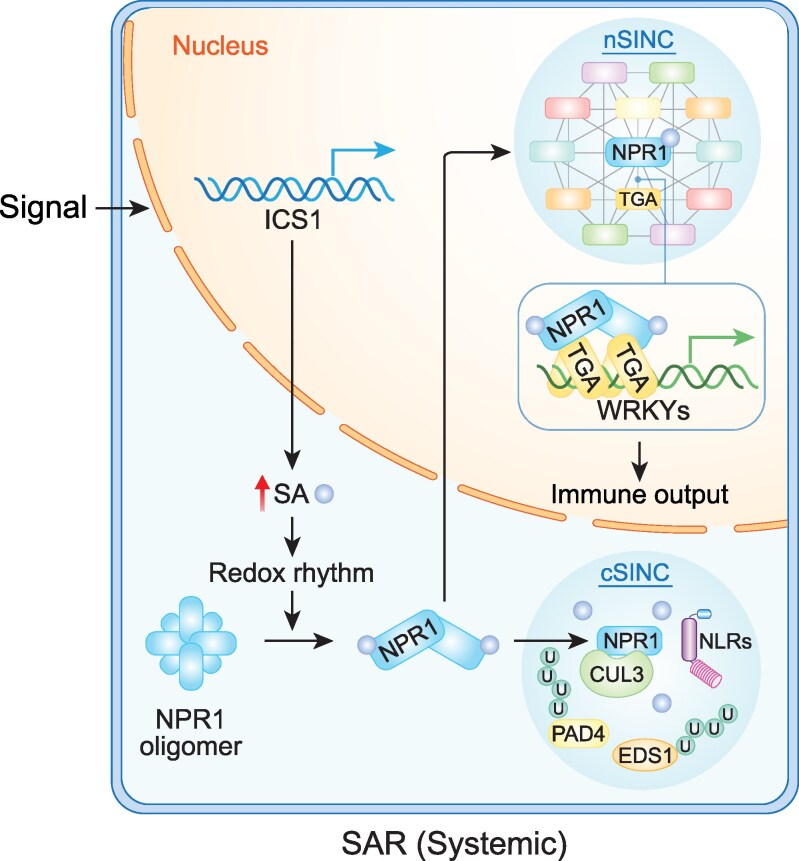
NPR1 forms distinct biomolecular condensates upon SA induction through perturbation of cellular redox. In response to SA induction, NPR1 can first form nSINCs containing general transcriptional regulatory complexes to activate the transcriptional cascade through a direct association with TGA TFs leading to the expression of secondary TF genes, such as *WRKY*s, to induce SAR output genes. Later during the SA induction, NPR1 can also form cytoplasmic condensates (cSINCs), containing many NB-LRRs (NLRs) and their signaling components such as EDS1 and PAD4, as well as other stress proteins to sequester or degrade defense proteins to resolve immune responses through the activities of protein degradation machineries, such as CUL3, and autophagosome components also found in these condensates. This figure was generated by Dr. Y. Xiang using BioRender.

My lab had been studying the NPR1 protein for more than 2 decades before we finally solved the protein structures with the advancement of the cryo-electron microscopy (cryo-EM) technology (Table 1). The active NPR1 turned out to be a dimer that looks like a gliding bird. When Pei Zhou, my collaborator at Duke Medical Center, showed me the beautiful cryo-EM images, it took my breath away. The journey to solve the NPR1 structure began with a meeting I had with Ning Zheng, a leading structural biologist renowned for solving the structures of receptors for the plant hormones, auxin and JA. He showed that perception of these hormones involves hormone-dependent complex formation between F-box proteins with their respective substrate transcription repressors leading to their degradation (Tan et al. 2007; Sheard et al. 2010). At the 2011 International Conference on Arabidopsis Research, Ning and I discussed the possibility of NPR1 being regulated in a similar manner because NPR1 contains a Broad-complex, Tramtrack, and Bric-à-brac (BTB)-domain, which has been shown to be present in many Cullin3 ubiquitin ligase adaptor proteins (van den Heuvel 2004). However, we neither knew the substrate for NPR1, nor observed a significant level of NPR1 binding to SA. As an alternative, we solved the structure of the SA-binding domain (SBD) of NPR4, a NPR1 paralog that has a much higher SA-binding affinity (Fu et al. 2012; Wang et al. 2020). I then took the opportunity to collaborate with Pei Zhou when Duke University called for projects to test our newly established cryo-EM facility. Even in the cryo-EM images of the full-length NPR1 (Kumar et al. 2022), the C-terminal SA-binding domain had to be modeled based on the NPR4 SBD structure (Wang et al. 2020), because the region is disordered in the absence of SA. To explain how NPR1 functions as a dimer, we also solved the structure of NPR1 in complex with the TGA3 TF, which showed that the NPR1 dimer crosslinks 2 DNA-bound TGA3 dimers to form an enhanceosome (Kumar et al. 2022) (Fig. 3). However, why SA-mediated docking of the SBD to the ankyrin repeat domain is required for NPR1 activity remains a mystery.

To identify the components of the NPR1-enhanceosome, we used a recently developed proximity labeling technique using TurboID-based biotinylation, followed by label-free quantification mass spectrometry (MS) (Powers et al. 2024) (Table 1). Surprisingly, we found that the NPR1-proxiome has a striking similarity to the GUANYLATE-BINDING PROTEIN-LIKE 3-proxiome, which promotes SA synthesis (Huang et al. 2021; Kim et al. 2022; Tang et al. 2022). Both proxiome share a similar set of general transcriptional complexes and regulators, except for the associated TFs, suggesting that common regulatory modules are recruited to reprogram specific transcriptomes by transcriptional cofactors, like NPR1, through binding to unique TFs (Fig. 3).

To determine whether NPR1 reprograms SA-induced transcriptome through parallel interactions with distinct TFs or a sequential transcriptional cascade, we adapted, for plants, the green fluorescent protein-tagged factor cleavage under target and release using nuclease (greenCUT&RUN) technique, which maps protein binding to DNA with significantly increased sensitivity than other methodologies (Koidl and Timmers 2021) (Table 1). Through stepwise greenCUT&RUN, we found that, upon SA induction, NPR1 initiates the transcriptional cascade primarily through association with TGA TFs to induce the expression of secondary TFs, predominantly WRKYs, which control immune-output genes without interacting with NPR1 at the chromatin (Powers et al. 2024). The finding that WRKYs act as secondary TFs for SA-induced output gene expression clarified for me why SA-induced transcriptomic data consistently showed enrichment of W-box for WRKY TFs, rather than the *as-1* element for the TGA TFs (Maleck et al. 2000; Wang et al. 2006; Ding et al. 2018; Jin et al. 2018; Powers et al. 2024). It appears that the small number of first tier TGA-targets, encoding mainly TFs, were masked in the statistical analyses by the larger number of WRKY-targets mainly encoding enzymes, a caveat that one needs to consider when interpreting results from large dataset analyses.

Besides the SA-induced NPR1-enhanceosome, which could be visualized as nuclear condensates (nSINCs) through GFP-tagging, NPR1-GFP could also be detected as cytoplasmic condensates at a higher concentration of SA (cSINCs), which were found to contain a large number of NB-LRR receptor proteins, their downstream signaling molecules, such as EDS1, and those involved in protein degradation and autophagy (Zavaliev et al. 2020) (Fig. 3). These data indicate that besides activating defense gene transcription, NPR1 can also sequester or degrade defense proteins to resolve immune responses, perhaps acting as a Cullin 3 adaptor protein, as suggested by its BTB-domain.

There are several questions that remain to be answered: (i) How does NPR1 form distinct biomolecular condensates? (ii) What are functional complexes within or in addition to those in the condensates? (iii) What are the dynamics in forming these complexes and condensates? Although this emerging field of studying biomolecular condensates is fascinating, it awaits major technological advances to overcome current limitations, such as the reliance on overexpression for detection and the lack of understanding of constituents as well as internal organization and dynamics.

## Precision in expressing plant immunity: not lost in translation

In the more than 3 decades of studying immune mechanisms, applying our new knowledge to improve plant disease resistance in agriculture has always been in the back of my mind. Specifically, I believe that broad-spectrum disease resistance is a good alternative to the NB-LRR-mediated ETI even though the latter has been widely used in agricultural practice for a long time, through breeding (Mapuranga et al. 2022), transgenic technology (Tai et al. 1999; Luo et al. 2021), and more recently, the Clustered Regulatory Interspaced Short Palindromic Repeats (CRISPR)-based approach (Li et al. 2024; Sun et al. 2024). However, the high level of signal specificity through the “gene-for-gene” mechanism introduces uncertainty in its commercial application. It is much like asking people to buy health insurance for 1 disease at a time. Instead, the use of broad-spectrum antibiotics has been so successful in controlling infectious diseases in humans that it has extended the average life expectancy by 23 yr (Hutchings et al. 2019). However, the major challenges in engineering broad-spectrum disease resistance in crops have been the associated fitness costs when defense genes are expressed constitutively. For example, overexpressing the *Arabidopsis NPR1* gene in many crop species has been found to enhance resistance to a wide variety of bacterial, fungal, viral pathogens, and even pests (Silva et al. 2018). However, this strategy has not been widely applied in agriculture, at least in part due to associated yield penalties.

I asked the question: How do plants naturally manage to have intrinsic broad-spectrum resistance mechanisms? From our study of SA-mediated induction of NPR1, we learned that this is accomplished through multiple layers of regulation described above: the antagonistic regulation of NPR1 by the central nutrient sensors TOR and SnRK1. NPR1 not only turns on transcription of *PR* and *ER* genes, but also feedback-represses SA synthesis (Shah et al. 1997; Wang et al. 2006) and becomes unstable in the nucleus to undergo proteasome-mediated degradation (Spoel et al. 2009). NPR1 also accumulates in the cytoplasm, at later time point, to remove a large number of defense proteins (eg NB-LRRs) all at once through the formation of SA-induced NPR1 condensates (ie cSINCs) to resolve the immune response (Zavaliev et al. 2020). However, even a highly versatile, multifunctional immune protein such as NPR1 may have been inadvertently subjected to negative selection during crop breeding for high yield, since defense traits often incur a cost to productivity. Therefore, to engineer broad-spectrum disease resistance without compromising yield, we need to find a way to have the best of both worlds.

A breakthrough in addressing this defense-vs.-yield conundrum came during our investigation into how SA treatment orchestrates the coordinated activation of defense-related genes, including the *ER* genes, while repressing the growth-related gene expression by the TL1-element binding factor (TBF1) TF (Pajerowska-Mukhtar et al. 2012). Since TBF1 is a heat shock factor derivative, we tried to investigate the possibility that it is regulated through multimer formation upon induction, like the human heat shock factor in response to heat treatment (Sarge et al. 1993). The difficulty that we encountered in obtaining transgenic lines overexpressing the TBF1 coding sequence (CDS) indicated that the protein might be too toxic to the plants when expressed constitutively. Using the pre-genomic era technology “5′ race” assay to define the 5′ end of the mRNA, we discovered that the *TBF1* mRNA contains not only the *TBF1* CDS but also 2 upstream open reading frames (uORFs) in the 5′ leader sequence (5′LS). Based on my molecular biology training, this finding was unusual. Normally, eukaryotic translation starts at the 5′ cap with the translation initiation complex scanning the transcript until it reaches the first AUG to begin translation. The 2 uORFs in the 5′LS of the *TBF1* mRNA would trap the ribosome from reaching the downstream main AUG (mAUG) for TBF1 translation. This turned out to be the case, because deleting the upstream AUGs (uAUGs) indeed allowed high TBF1 expression in transient assays in *N. benthamiana* leaves, which resulted in massive cell death in the inoculated leaves (Xu et al. 2017b).

We were not the first to discover uORF regulation of stress-responsive TFs. In yeast and mammals, translation of TFs, such as GCN4 and ATF4, is normally inhibited by the presence of uORFs (Sonenberg and Hinnebusch 2009). However, upon amino acid starvation and other stresses, the presence of uncharged tRNAs serves as a signal to activate the GCN2 kinase which phosphorylates the eukaryotic translation initiation factor eIF2α required for initiating translation. The resulting p-eIF2α is less active in supporting global translation or initiation at uAUGs, allowing the initiation complex to reach downstream mAUGs to translate the stress-responsive TFs. Surprisingly, when we tested TBF1 translation in the *Arabidopsis gcn2* knockout mutant, we found that while phosphorylation of eIF2α was blocked, induction of TBF1 translation was not affected (Xu et al. 2017a). The data indicated that there are new stress-responsive translational regulatory mechanisms to be discovered. The conservation of the highly toxic *TBF1* gene across a wide range of plant species (Hayden and Jorgensen 2007; Xu et al. 2017b) suggests that its full-length mRNA contains regulatory elements that can tightly control protein translation, keeping it repressed under normal conditions, and allowing transient expression only upon pathogen challenge.

Without knowing the mechanisms, we used the 5′ LS sequence of *TBF1* to drive the translation of the *Arabidopsis* autoactive *NB-LRR* gene, *snc1* (Zhang et al. 2003), and found that it could indeed avoid the dwarf phenotype caused by constitutively expressing the *snc1* mutant, while maintaining the enhanced resistance against bacterial and oomycete pathogens (Xu et al. 2017b). Encouraged by this initial success, we extended the approach to rice in collaboration with Dr. Shiping Wang's lab. By putting the NPR1 CDS under the control of the *TBF1* 5′ LS, we were able to maintain WT levels of growth and yield for the transgenic plants in the field while enhancing resistance to rice bacterial blight and fungal blast (Xu et al. 2017b), providing the proof of concept for using “the TBF1-switchblade” in engineering broad-spectrum disease resistance without a yield penalty.

The success in the rice field pulled us back into the lab to understand the underlying mechanisms that regulate defense protein production. The development of ribosome profiling (Ribo-seq) provided a great opportunity for surveying global translatomic changes using the ratio of ribosome-associated mRNA to total mRNA as a proxy for measuring translation efficiency (TE) (Ingolia et al. 2009) (Table 1). From our Ribo-seq analyses of pattern-triggered immunity (PTI) and ETI, we found that PTI-associated translational changes, which occurred within 1 h of treatment with microbe-associated molecular patterns, were poorly correlated with the transcriptional changes (Xu et al. 2017a), while a good correlation was observed 8 h after ETI-induction (Yoo et al. 2020), consistent with our prediction that translation is more likely to be distinctly regulated from preexisting transcripts during PTI, a fast response which is the first line of active plant defense, instead of ETI, which occurs later during infection.

**Figure 4.**
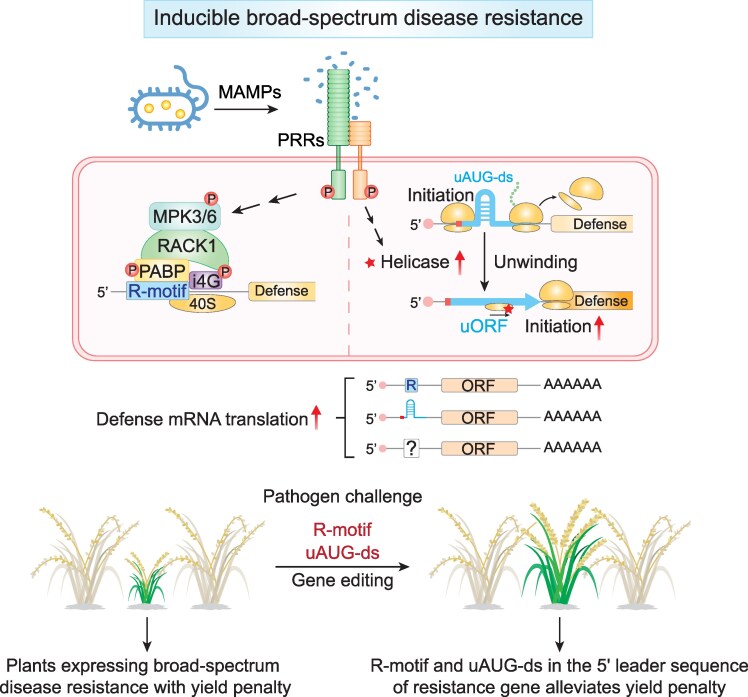
Translational regulatory modules involved in controlling defense protein translation can be used as molecular switches for engineering broad-spectrum disease-resistant crops in agriculture. Through Ribo-seq, an enriched *cis*-element containing only purine residues (“R-motif”) has been found in the 5′ LSs of transcripts with PTI-induced increase in translational efficiency. The R-motif serves as a cellular IRES through recruitment of translation initiation components, PABPs, and eIFiso4G, after both have been phosphorylated by the immune regulators, MPK3/6, recruited by the chaperone protein RACK1, to activate immune protein translation independent of the 5′-cap. Beside the positive element, R-motif, transcripts with immune-induced translation are also enriched with uORFs. Under normal conditions, the selective translation is determined by double-stranded structures located immediately downstream of the uAUGs (“uAUG-ds”). These structures facilitate the engagement of the initiation complex, enabling efficient recruitment of the 60S ribosome to begin translating uORF instead of downstream defense gene translation. Upon pathogen challenge, RNA helicases are required for unwinding uAUG-ds structures to alleviate repression on defense protein translation. R-motif and uAUG-ds are molecular switches that can be used to make translation of immune regulators pathogen-inducible to enhance broad-spectrum disease resistance in crops while minimizing the fitness penalty that is often associated with constitutive expression of defense genes. This figure was generated by Y. Xiang using BioRender.

From our Ribo-seq data, we identified an enriched *cis*-element in the 5′ LSs of transcripts with PTI-induced increases in TE (including *TBF1*) that contained only purine residues, which we named “R-motif” (Xu et al. 2017a) (Fig. 4). Mechanistically, we showed that the R-motif serves as a cellular internal ribosome entry site (IRES) through recruitment of translation initiation components, poly(A)-binding proteins (PABPs) and eIFiso4G, after both have been phosphorylated by the known central immune regulators, MPK3/6 (Wang et al. 2022) (Fig. 4). R-motif sequences have also been found as IRES-like elements in yeast in starvation-induced differentiation (Gilbert et al. 2007) and in HEK293T cells in a screen for IRES-like elements (Fan et al. 2022). Since we have shown that the R-motif is not only necessary but also sufficient for PTI-induced translation (Xu et al. 2017a), it represents an ideal regulatory module for engineering PTI-induced protein production in plants and, possibly, in other eukaryotes for synthesis of general stress-responsive proteins.

With a positive *cis*-element (ie R-motif) in hand, we next studied the negative arm of the regulation, ie how ribosomes selectively translate uAUGs to inhibit mORF translation in the absence of pathogen challenge. It is unlikely that all uAUGs can initiate translation as they are far more prevalently present in the 5′LSs of transcripts than previously anticipated (54.6% of the transcripts in *Arabidopsis* and 64.1% in humans) (Zhang et al. 2021). To answer this fundamental biological question, we adapted another new technology known as SHAPE-MaP (selective 2′-hydroxyl acylation analyzed by primer extension and mutational profiling) (Siegfried et al. 2014) to profile in vivo mRNA secondary structural changes upon PTI induction (Xiang et al. 2023) (Table 1). We observed that transcripts with immune-induced translation are enriched with uORFs, and their selective translation under normal conditions is determined by double-stranded structures located immediately downstream of the uAUGs (we named “uAUG-ds”). These structures facilitate the engagement of the initiation complex, enabling efficient recruitment of the 60S ribosome to begin translation (Fig. 4). Deep learning analyses of the Ribo-seq and SHAPE-MaP data in collaboration with Qiangfeng Zhang's lab led to the development of “TISnet” algorithm to define optimal ranges of the hairpin length and folding energy for these structures in dictating translation initiation for not only uAUGs but also mAUGs. The accuracy of the prediction has been comprehensively tested in plants as well as in a human cell line, HEK293FT (Xiang et al. 2023). Moreover, we have also identified immune-induced RNA helicase RH37 and its homologs RH11 and RH52 to be required for unwinding uAUG-ds structures upon PTI induction, which is blocked in their higher-order mutants (Xiang et al. 2023) (Fig. 4). RH11/37/52 is homologous to Ded1p in yeast and DDX3X in humans known to be associated with the initiation complex in regulating translation initiation (Sen et al. 2015; Ryan and Schroder 2022).

With uAUG-ds defined by hairpin length and folding energy and the identification of corresponding helicases (Xiang et al. 2023), we now have another tunable translational module, besides R-motif, to control the levels of defense protein translation by applying the CRISPR-based gene editing tools (Gupta et al. 2023, 2024; Li et al. 2024; Sun et al. 2024) to engineer broad-spectrum disease resistance in agriculture (Fig. 4).

## Getting down to the business of engineering broad-spectrum disease resistance

My interest in applying what we discovered in the lab to agricultural applications is shared by my former graduate student, George Greene, who was involved in identifying the R-motif in the PTI-responsive transcripts (Xu et al. 2017a). The discovery and characterization of the translational regulatory sequences and structures presented an opportunity to “translate” basic research into real-world applications, specifically, by developing pathogen-inducible broad-spectrum disease-resistant crops without compromising yield. To expose ourselves to the business world, we enrolled in the NSF Innovation Corps (I-Corps), which is designed to help NSF grantees transform their scientific discoveries into commercial products. It provided 7 wk of basic training in the areas of entrepreneurship, business, and product commercialization. These 7 wk led to 7 yr of building our startup company, Upstream Biotechnology, Inc., through which I have witnessed George's metamorphosis into a self-confident and effective CEO who grew his connections in the ag-business sector from ground zero. I asked George what it took to keep him going all these years. His answer was optimism and perseverance. These are also elements for success in basic research and life in general. But life in academia and industry is very different. In academia, you can impress people with your publications and seminars without having to get to know them in person. However, in business, connections are critical. For Upstream, we are quite fortunate to have the help of John Salmeron, who studied plant resistance mechanisms when the field was in its infancy (Salmeron et al. 1996) and has decades of ag-business experience. A person who can translate between academia and industry is essential because we do not speak the same language. But training in performing scientific research has taught us the ability to explore the unexplored and learn new skills with optimism and perseverance.

## Reflection on the past and outlook for the future

The reviewer suggested that I add a few sentences to this article to highlight the impacts of my past work so that young readers can learn how to identify important scientific questions that have broad impacts. I found this a challenging task because my view of my work sways from “tedious” to “groundbreaking.” This is why I continue to teach undergraduates: I can be certain that I am making an impact on my students’ lives. In fact, scientific research is full of tedious grunt work that might someday lead to groundbreaking discoveries. Our interest in the research topic has to be sufficiently high to sustain us through the hard work and failures. My initial fascination with how a local infection can lead to systemic broad-spectrum resistance in plants presented many fundamental questions for me to answer. The identification of NPR1 as a master immune regulator, with its direct connection to the circadian clock and central metabolic sensor kinases, opened the door for me to investigate how environmental cues, cellular metabolism, and redox status impact immunity. My desire to apply broad-spectrum resistance in agriculture led us to investigate the fundamental question how stress protein translation is regulated.

To tackle “big” scientific questions, the necessary technologies must be in place. With the rapid pace of technological advancement, there is still much I hope to accomplish in my research career. I would like to understand the dynamics of NPR1 condensates in the nucleus and the cytoplasm that enable it to perform its distinct functions. This will probably involve breakthroughs in live cell imaging technologies as well as methods, such as cross-linking MS (Yu and Huang 2023) that can provide information at structural, molecular, and systems biology levels. Regarding translational regulation, we have only scratched the surface. Beyond identifying 2 regulatory modules for defense-induced translational initiation, we would like to understand the regulatory mechanisms at the elongation step, which includes mRNA modifications, stability, and subcellular localization, tRNA availability, and modifications in the ribosome (Xiang and Dong 2025). Once again, imaging of the translational process at single mRNA (Khuperkar et al. 2020) and whole translatome levels will significantly increase the resolution of our studies. The current Ribo-seq protocol is still quite cumbersome and expensive. High-throughput Ribo-seq would allow a much-needed general survey of many plant species and in more stress responses to discover common and distinct regulatory mechanisms. My ongoing interest in the cellular redox and the circadian clock has been further heightened by our recent discovery that the central metabolic regulators, SnRK1 and TOR, are kinases that antagonistically phosphorylate NPR1 upon SA induction, making a direct link between plant defense and metabolic activities (Chen et al. 2025). I may not have the expertise, at this moment, to make a technological wish list for our future study of defense–metabolism relationship, but as always, the technical challenges and the solutions to overcome them will emerge in due time. I am also optimistic that we are getting closer to realizing the dream of engineering broad-spectrum disease resistance for crop protection in agriculture.

## Data Availability

This review contains no original data.

## Dive Curated Terms

The following phenotypic, genotypic, and functional terms are of significance to the work described in this paper:

NPR4 Gramene: AT4G19660
NPR4 Araport: AT4G19660

NSF Gramene: AT4G04910
NSF Araport: AT4G04910

RIN4 Gramene: AT3G25070
RIN4 Araport: AT3G25070

RPM1 Gramene: AT3G07040
RPM1 Araport: AT3G07040

EDS1 Gramene: AT3G48090
EDS1 Araport: AT3G48090

BGL2 Gramene: AT3G57260
BGL2 Araport: AT3G57260

PR1 Gramene: AT2G14610
PR1 Araport: AT2G14610

CCA1 Gramene: ostta06g02340
CCA1 Araport: ostta06g02340

TGA3 Gramene: AT1G22070
TGA3 Araport: AT1G22070

RPP4 Gramene: AT4G16860
RPP4 Araport: AT4G16860

NPR1 Gramene: AT1G64280
NPR1 Araport: AT1G64280

SNC1 Gramene: AT4G16890
SNC1 Araport: AT4G16890

GCN2 Gramene: At3g59410
GCN2 Araport: At3g59410

## References

[kiaf615-B1] Abruzzi KC, Gobet C, Naef F, Rosbash M. Comment on “Circadian rhythms in the absence of the clock gene *Bmal1*”. Science. 2021:372:eabe9230. 10.1126/science.abf0922. 33859000

[kiaf615-B2] Ahuja I, Kissen R, Bones AM. Phytoalexins in defense against pathogens. Trends Plant Sci. 2012:17:73–90. 10.1016/j.tplants.2011.11.002. 22209038

[kiaf615-B3] Backer R, Naidoo S, van den Berg N. The nonexpressor of pathogenesis-related genes 1 (NPR1) and related family: mechanistic insights in plant disease resistance. Front Plant Sci. 2019:10:102. 10.3389/fpls.2019.00102. 30815005 PMC6381062

[kiaf615-B4] Bent AF et al RPS2 of *Arabidopsis thaliana*: a leucine-rich repeat class of plant disease resistance genes. Science. 1994:265:1856–1860. 10.1126/science.8091210. 8091210

[kiaf615-B5] Bhardwaj V, Meier S, Petersen LN, Ingle RA, Roden LC. Defence responses of *Arabidopsis thaliana* to infection by *Pseudomonas syringae* are regulated by the circadian clock. PLoS One. 2011:6:e26968. 10.1371/journal.pone.0026968. 22066021 PMC3205005

[kiaf615-B6] Bonardi V et al Expanded functions for a family of plant intracellular immune receptors beyond specific recognition of pathogen effectors. Proc Natl Acad Sci U S A. 2011:108:16463–16468. 10.1073/pnas.1113726108. 21911370 PMC3182704

[kiaf615-B7] Bowling SA et al A mutation in Arabidopsis that leads to constitutive expression of systemic acquired resistance. Plant Cell. 1994:6:1845–1857. 10.1105/tpc.6.12.1845. 7866028 PMC160566

[kiaf615-B8] Cao H, Bowling SA, Gordon AS, Dong X. Characterization of an Arabidopsis mutant that is nonresponsive to inducers of systemic acquired resistance. Plant Cell. 1994:6:1583–1592. 10.2307/3869945. 12244227 PMC160545

[kiaf615-B9] Cao H, Glazebrook J, Clarke JD, Volko S, Dong X. The Arabidopsis NPR1 gene that controls systemic acquired resistance encodes a novel protein containing ankyrin repeats. Cell. 1997:88:57–63. 10.1016/S0092-8674(00)81858-9. 9019406

[kiaf615-B10] Cao L et al H_2_O_2_ sulfenylates CHE, linking local infection to the establishment of systemic acquired resistance. Science. 2024:385:1211–1217. 10.1126/science.adj7249. 39265009 PMC11586058

[kiaf615-B11] Chen Y et al Salicylic acid engages central metabolic regulators SnRK1 and TOR to govern immunity by differential phosphorylation of NPR1. bioRxiv. 10.1101/2025.06.17.660129.

[kiaf615-B12] Chen Z, Silva H, Klessig DF. Active oxygen species in the induction of plant systemic acquired resistance by salicylic acid. Science. 1993:262:1883–1886. 10.1126/science.8266079. 8266079

[kiaf615-B13] Dangl JL, Jones JD. Plant pathogens and integrated defence responses to infection. Nature. 2001:411:826–833. 10.1038/35081161. 11459065

[kiaf615-B14] Delaney TP, Friedrich L, Ryals JA. Arabidopsis signal transduction mutant defective in chemically and biologically induced disease resistance. Proc Natl Acad Sci U S A. 1995:92:6602–6606. 10.1073/pnas.92.14.6602. 11607555 PMC41566

[kiaf615-B15] Derevnina L, Kamoun S, Wu CH. Dude, where is my mutant? *Nicotiana benthamiana* meets forward genetics. New Phytol. 2019:221:607–610. 10.1111/nph.15521. 30569612

[kiaf615-B16] Després C, DeLong C, Glaze S, Liu E, Fobert PR. The Arabidopsis NPR1/NIM1 protein enhances the DNA binding activity of a subgroup of the TGA family of bZIP transcription factors. Plant Cell. 2000:12:279–290. 10.1105/tpc.12.2.279. 10662863 PMC139764

[kiaf615-B17] Dewdney J et al Three unique mutants of *Arabidopsis* identify eds loci required for limiting growth of a biotrophic fungal pathogen. Plant J. 2000:24:205–218. 10.1046/j.1365-313x.2000.00870.x. 11069695

[kiaf615-B18] Ding Y et al Opposite roles of salicylic acid receptors NPR1 and NPR3/NPR4 in transcriptional regulation of plant immunity. Cell. 2018:173:1454–1467.e15. 10.1016/j.cell.2018.03.044. 29656896

[kiaf615-B19] Dong X, Mindrinos M, Davis KR, Ausubel FM. Induction of Arabidopsis defense genes by virulent and avirulent *Pseudomonas syringae* strains and by a cloned avirulence gene. Plant Cell. 1991:3:61–72. 10.1105/tpc.3.1.61. 1824335 PMC159979

[kiaf615-B20] Dong X, Womble DD, Luckow VA, Rownd RH. Regulation of transcription of the repA1 gene in the replication control region of IncFII plasmid NR1 by gene dosage of the repA2 transcription repressor protein. J Bacteriol. 1985:161:544–551. 10.1128/jb.161.2.544-551.1985. 3155722 PMC214916

[kiaf615-B21] Dong XN, Rouillard KP, Womble DD, Rownd RH. DNA bending near the replication origin of IncFII plasmid NR1. J Bacteriol. 1989:171:703–707. 10.1128/jb.171.2.703-707.1989. 2644234 PMC209654

[kiaf615-B22] Dong XN, Womble DD, Rownd RH. Transcriptional pausing in a region important for plasmid NR1 replication control. J Bacteriol. 1987:169:5353–5363. 10.1128/jb.169.12.5353-5363.1987. 2445727 PMC213958

[kiaf615-B23] Dong XN, Womble DD, Rownd RH. In-vivo studies on the cis-acting replication initiator protein of IncFII plasmid NR1. J Mol Biol. 1988:202:495–509. 10.1016/0022-2836(88)90281-1. 3050127

[kiaf615-B24] Edgar RS et al Peroxiredoxins are conserved markers of circadian rhythms. Nature. 2012:485:459–464. 10.1038/nature11088. 22622569 PMC3398137

[kiaf615-B25] Fan X, Yang Y, Chen C, Wang Z. Pervasive translation of circular RNAs driven by short IRES-like elements. Nat Commun. 2022:13:3751. 10.1038/s41467-022-31327-y. 35768398 PMC9242994

[kiaf615-B26] Flor HH . Inheritance of pathogenicity in *Melampsora lini*. Phytopathology. 1942:32:653–669. 10.1094/Phyto-32-653. 40934406

[kiaf615-B27] Flor HH . Current status of the gene-for-gene concept. Annu Rev Phytopathol. 1971:9:275–296. 10.1146/annurev.py.09.090171.001423.

[kiaf615-B28] Frederick RD, Thilmony RL, Sessa G, Martin GB. Recognition specificity for the bacterial avirulence protein AvrPto is determined by Thr-204 in the activation loop of the tomato Pto kinase. Mol Cell. 1998:2:241–245. 10.1016/S1097-2765(00)80134-3. 9734361

[kiaf615-B29] Friedrich L et al A benzothiadiazole derivative induces systemic acquired resistance in tobacco. Plant J. 1996:10:61–70. 10.1046/j.1365-313X.1996.10010061.x. 8758979

[kiaf615-B30] Fu ZQ et al NPR3 and NPR4 are receptors for the immune signal salicylic acid in plants. Nature. 2012:486:228–232. 10.1038/nature11162. 22699612 PMC3376392

[kiaf615-B31] Gaffney T et al Requirement of salicylic acid for the induction of systemic acquired resistance. Science. 1993:261:754–756. 10.1126/science.261.5122.754. 17757215

[kiaf615-B32] Gilbert WV, Zhou K, Butler TK, Doudna JA. Cap-independent translation is required for starvation-induced differentiation in yeast. Science. 2007:317:1224–1227. 10.1126/science.1144467. 17761883

[kiaf615-B33] Glazebrook J, Rogers EE, Ausubel FM. Isolation of Arabidopsis mutants with enhanced disease susceptibility by direct screening. Genetics. 1996:143:973–982. 10.1093/genetics/143.2.973. 8725243 PMC1207353

[kiaf615-B34] Goodin MM, Zaitlin D, Naidu RA, Lommel SA. *Nicotiana benthamiana*: its history and future as a model for plant-pathogen interactions. Mol Plant Microbe Interact. 2015:2015:28–39. 10.1094/MPMI-00-00-1015-REV.testissue. 27839076

[kiaf615-B35] Goodspeed D, Chehab EW, Covington MF, Braam J. Circadian control of jasmonates and salicylates: the clock role in plant defense. Plant Signal Behav. 2013:8:e23123. 10.4161/psb.23123. 23299428 PMC3657008

[kiaf615-B36] Goodspeed D, Chehab EW, Min-Venditti A, Braam J, Covington MF. Arabidopsis synchronizes jasmonate-mediated defense with insect circadian behavior. Proc Natl Acad Sci U S A. 2012:109:4674–4677. 10.1073/pnas.1116368109. 22331878 PMC3311395

[kiaf615-B37] Gorina S, Pavletich NP. Structure of the p53 tumor suppressor bound to the ankyrin and SH3 domains of 53BP2. Science. 1996:274:1001–1005. 10.1126/science.274.5289.1001. 8875926

[kiaf615-B38] Gorlach J et al Benzothiadiazole, a novel class of inducers of systemic acquired resistance, activates gene expression and disease resistance in wheat. Plant Cell. 1996:8:629–643. 10.1105/tpc.8.4.629. 8624439 PMC161125

[kiaf615-B39] Gupta A, Liu B, Chen QJ, Yang B. High-efficiency prime editing enables new strategies for broad-spectrum resistance to bacterial blight of rice. Plant Biotechnol J. 2023:21:1454–1464. 10.1111/pbi.14049. 37139586 PMC10281596

[kiaf615-B40] Gupta A, Liu B, Raza S, Chen QJ, Yang B. Modularly assembled multiplex prime editors for simultaneous editing of agronomically important genes in rice. Plant Commun. 2024:5:100741. 10.1016/j.xplc.2023.100741. 37897041 PMC10873889

[kiaf615-B41] Hayden CA, Jorgensen RA. Identification of novel conserved peptide uORF homology groups in Arabidopsis and rice reveals ancient eukaryotic origin of select groups and preferential association with transcription factor-encoding genes. BMC Biol. 2007:5:32. 10.1186/1741-7007-5-32. 17663791 PMC2075485

[kiaf615-B42] He P, Shan L, Sheen J. The use of protoplasts to study innate immune responses. Methods Mol Biol. 2007:354:1–9. 10.1385/1-59259-966-4:1. 17172739

[kiaf615-B43] Hevia MA, Canessa P, Müller-Esparza H, Larrondo LF. A circadian oscillator in the fungus *Botrytis cinerea* regulates virulence when infecting *Arabidopsis thaliana*. Proc Natl Acad Sci U S A. 2015:112:8744–8749. 10.1073/pnas.1508432112. 26124115 PMC4507220

[kiaf615-B44] Huang S, Zhu S, Kumar P, MacMicking JD. A phase-separated nuclear GBPL circuit controls immunity in plants. Nature. 2021:594:424–429. 10.1038/s41586-021-03572-6. 34040255 PMC8478157

[kiaf615-B45] Hutchings MI, Truman AW, Wilkinson B. Antibiotics: past, present and future. Curr Opin Microbiol. 2019:51:72–80. 10.1016/j.mib.2019.10.008. 31733401

[kiaf615-B46] Ingolia NT, Ghaemmaghami S, Newman JR, Weissman JS. Genome-wide analysis in vivo of translation with nucleotide resolution using ribosome profiling. Science. 2009:324:218–223. 10.1126/science.1168978. 19213877 PMC2746483

[kiaf615-B47] Jefferson RA, Kavanagh TA, Bevan MW. GUS fusions: beta-glucuronidase as a sensitive and versatile gene fusion marker in higher plants. EMBO J. 1987:6:3901–3907. 10.1002/j.1460-2075.1987.tb02730.x. 3327686 PMC553867

[kiaf615-B48] Jin H et al Salicylic acid-induced transcriptional reprogramming by the HAC-NPR1-TGA histone acetyltransferase complex in Arabidopsis. Nucleic Acids Res. 2018:46:11712–11725. 10.1093/nar/gky847. 30239885 PMC6294559

[kiaf615-B49] Jones DA, Thomas CM, Hammond-Kosack KE, Balint-Kurti PJ, Jones JD. Isolation of the tomato Cf-9 gene for resistance to *Cladosporium fulvum* by transposon tagging. Science. 1994:266:789–793. 10.1126/science.7973631. 7973631

[kiaf615-B50] Karapetyan S, Mwimba M, Chen T, Yao Z, Dong X. The redox rhythm gates immune-induced cell death distinctly from the genetic clock. Proc Natl Acad Sci U S A. 2025:122:e2519251122. 10.1073/pnas.2519251122. 40928881 PMC12452835

[kiaf615-B51] Keen NT, Yoshikawa M. β-1,3-Endoglucanase from soybean releases elicitor-active carbohydrates from fungus cell walls. Plant Physiol. 1983:71:460–465. 10.1104/pp.71.3.460. 16662849 PMC1066060

[kiaf615-B52] Khuperkar D et al Quantification of mRNA translation in live cells using single-molecule imaging. Nat Protoc. 2020:15:1371–1398. 10.1038/s41596-019-0284-x. 32076351

[kiaf615-B53] Kim HS, Delaney TP. Over-expression of *TGA5*, which encodes a bZIP transcription factor that interacts with NIM1/NPR1, confers SAR-independent resistance in *Arabidopsis thaliana* to *Peronospora parasitica*. Plant J. 2002:32:151–163. 10.1046/j.1365-313X.2001.01411.x. 12383081

[kiaf615-B54] Kim JH et al Increasing the resilience of plant immunity to a warming climate. Nature. 2022:607:339–344. 10.1038/s41586-022-04902-y. 35768511 PMC9279160

[kiaf615-B55] Koidl S, Timmers HTM. greenCUT&RUN: efficient genomic profiling of GFP-tagged transcription factors and chromatin regulators. Curr Protoc. 2021:1:e266. 10.1002/cpz1.266. 34644460

[kiaf615-B56] Korneli C, Danisman S, Staiger D. Differential control of pre-invasive and post-invasive antibacterial defense by the Arabidopsis circadian clock. Plant Cell Physiol. 2014:55:1613–1622. 10.1093/pcp/pcu092. 24974385

[kiaf615-B57] Kumar S et al Structural basis of NPR1 in activating plant immunity. Nature. 2022:605:561–566. 10.1038/s41586-022-04699-w. 35545668 PMC9346951

[kiaf615-B58] Kus JV, Zaton K, Sarkar R, Cameron RK. Age-related resistance in Arabidopsis is a developmentally regulated defense response to *Pseudomonas syringae*. Plant Cell. 2002:14:479–490. 10.1105/tpc.010481. 11884688 PMC152926

[kiaf615-B59] Lawton KA et al Benzothiadiazole induces disease resistance in *Arabidopsis* by activation of the systemic acquired resistance signal transduction pathway. Plant J. 1996:10:71–82. 10.1046/j.1365-313X.1996.10010071.x. 8758979

[kiaf615-B60] Lemaitre B, Nicolas E, Michaut L, Reichhart JM, Hoffmann JA. The dorsoventral regulatory gene cassette spatzle/toll/cactus controls the potent antifungal response in *Drosophila* adults. Cell. 1996:86:973–983. 10.1016/S0092-8674(00)80172-5. 8808632

[kiaf615-B61] Levvy GA, Marsh CA. Synthesis of 4-methylumbelliferone β-D-glucuronide, a substrate for the fluorimetric assay of β-glucuronidase. Nature. 1956:178:589–590. 10.1038/178589b0. 13369469

[kiaf615-B62] Li B, Sun C, Li J, Gao C. Targeted genome-modification tools and their advanced applications in crop breeding. Nat Rev Genet. 2024:25:603–622. 10.1038/s41576-024-00720-2. 38658741

[kiaf615-B63] Li H, Qiu J, Fu XD. RASL-seq for massively parallel and quantitative analysis of gene expression. Curr Protoc Mol Biol. 2012:98:Unit 4.13.1–Unit 4.13.9. 10.1002/0471142727.mb0413s98. PMC3325489 22470064

[kiaf615-B64] Liu L et al Salicylic acid receptors activate jasmonic acid signalling through a non-canonical pathway to promote effector-triggered immunity. Nat Commun. 2016:7:13099. 10.1038/ncomms13099. 27725643 PMC5062614

[kiaf615-B65] Liu Y et al Three-step biosynthesis of salicylic acid from benzoyl-CoA in plants. Nature. 2025:645:201–207. 10.1038/s41586-025-09185-7. 40702178

[kiaf615-B66] Lu H, McClung CR, Zhang C. Tick tock: circadian regulation of plant innate immunity. Annu Rev Phytopathol. 2017:55:287–311. 10.1146/annurev-phyto-080516-035451. 28590878

[kiaf615-B67] Luo M et al A five-transgene cassette confers broad-spectrum resistance to a fungal rust pathogen in wheat. Nat Biotechnol. 2021:39:561–566. 10.1038/s41587-020-00770-x. 33398152

[kiaf615-B68] Mackey D, Belkhadir Y, Alonso JM, Ecker JR, Dangl JL. Arabidopsis RIN4 is a target of the type III virulence effector AvrRpt2 and modulates RPS2-mediated resistance. Cell. 2003:112:379–389. 10.1016/S0092-8674(03)00040-0. 12581527

[kiaf615-B69] Mackey D, Holt BF 3rd, Wiig A, Dangl JL. RIN4 interacts with *Pseudomonas syringae* type III effector molecules and is required for RPM1-mediated resistance in *Arabidopsis*. Cell. 2002:108:743–754. 10.1016/S0092-8674(02)00661-X. 11955429

[kiaf615-B70] Malamy J, Carr JP, Klessig DF, Raskin I. Salicylic acid: a likely endogenous signal in the resistance response of tobacco to viral infection. Science. 1990:250:1002–1004. 10.1126/science.250.4983.1002. 17746925

[kiaf615-B71] Maleck K et al The transcriptome of *Arabidopsis thaliana* during systemic acquired resistance. Nat Genet. 2000:26:403–410. 10.1038/82521. 11101835

[kiaf615-B72] Mapuranga J et al Harnessing genetic resistance to rusts in wheat and integrated rust management methods to develop more durable resistant cultivars. Front Plant Sci. 2022:13:951095. 10.3389/fpls.2022.951095. 36311120 PMC9614308

[kiaf615-B73] Mauch F, Hadwiger LA, Boller T. Antifungal hydrolases in pea tissue: I. Purification and characterization of two Chitinases and two β-1,3-glucanases differentially regulated during development and in response to fungal infection. Plant Physiol. 1988a:87:325–333. 10.1104/pp.87.2.325. 16666142 PMC1054752

[kiaf615-B74] Mauch F, Mauch-Mani B, Boller T. Antifungal hydrolases in pea tissue: II. Inhibition of fungal growth by combinations of Chitinase and beta-1,3-glucanase. Plant Physiol. 1988b:88:936–942. 10.1104/pp.88.3.936. 16666407 PMC1055685

[kiaf615-B75] Mauch F, Staehelin LA. Functional implications of the subcellular localization of ethylene-induced Chitinase and [β]-1,3-glucanase in bean leaves. Plant Cell. 1989:1:447–457. 10.2307/3869105. 12359894 PMC159776

[kiaf615-B76] Mercurio F et al IKK-1 and IKK-2: cytokine-activated IκB kinases essential for NF-κB activation. Science. 1997:278:860–866. 10.1126/science.278.5339.860. 9346484

[kiaf615-B77] Metraux JP et al Increase in salicylic acid at the onset of systemic acquired resistance in cucumber. Science. 1990:250:1004–1006. 10.1126/science.250.4983.1004. 17746926

[kiaf615-B78] Mindrinos M, Katagiri F, Yu GL, Ausubel FM. The *A. thaliana* disease resistance gene RPS2 encodes a protein containing a nucleotide-binding site and leucine-rich repeats. Cell. 1994:78:1089–1099. 10.1016/0092-8674(94)90282-8. 7923358

[kiaf615-B79] Moll P, Ante M, Seitz A, Reda T. QuantSeq 3′ mRNA sequencing for RNA quantification. Nat Methods. 2014:11:i–iii. 10.1038/nmeth.f.376.

[kiaf615-B80] Mou Z, Fan W, Dong X. Inducers of plant systemic acquired resistance regulate NPR1 function through redox changes. Cell. 2003:113:935–944. 10.1016/S0092-8674(03)00429-X. 12837250

[kiaf615-B81] Mukhtar MS et al Independently evolved virulence effectors converge onto hubs in a plant immune system network. Science. 2011:333:596–601. 10.1126/science.1203659. 21798943 PMC3170753

[kiaf615-B82] Nawrath C, Metraux JP. Salicylic acid induction-deficient mutants of Arabidopsis express *PR-2* and *PR-5* and accumulate high levels of camalexin after pathogen inoculation. Plant Cell. 1999:11:1393–1404. 10.1105/tpc.11.8.1393. 10449575 PMC144293

[kiaf615-B83] Oldroyd GE, Staskawicz BJ. Genetically engineered broad-spectrum disease resistance in tomato. Proc Natl Acad Sci U S A. 1998:95:10300–10305. 10.1073/pnas.95.17.10300. 9707642 PMC21503

[kiaf615-B84] O'Neill JS et al Circadian rhythms persist without transcription in a eukaryote. Nature. 2011:469:554–558. 10.1038/nature09654. 21270895 PMC3040569

[kiaf615-B85] O'Neill JS, Reddy AB. Circadian clocks in human red blood cells. Nature. 2011:469:498–503. 10.1038/nature09702. 21270888 PMC3040566

[kiaf615-B86] Pajerowska-Mukhtar KM et al The HSF-like transcription factor TBF1 is a major molecular switch for plant growth-to-defense transition. Curr Biol. 2012:22:103–112. 10.1016/j.cub.2011.12.015. 22244999 PMC3298764

[kiaf615-B87] Panstruga R, Parker JE, Schulze-Lefert P. SnapShot: plant immune response pathways. Cell. 2009:136:978 e971–978 973. 10.1016/j.cell.2009.02.020. 19269372

[kiaf615-B88] Parker JE et al Characterization of eds1, a mutation in Arabidopsis suppressing resistance to Peronospora parasitica specified by several different RPP genes. Plant Cell. 1996:8:2033–2046. 10.1105/tpc.8.11.2033. 8953768 PMC161332

[kiaf615-B89] Poltorak A et al Defective LPS signaling in C3H/HeJ and C57BL/10ScCr mice: mutations in Tlr4 gene. Science. 1998:282:2085–2088. 10.1126/science.282.5396.2085. 9851930

[kiaf615-B90] Powers J et al Next-generation mapping of the salicylic acid signaling hub and transcriptional cascade. Mol Plant. 2024:17:1558–1572. 10.1016/j.molp.2024.08.008. 39180213 PMC11540436

[kiaf615-B91] Raskin I, Turner IM, Melander WR. Regulation of heat production in the inflorescences of an *Arum* lily by endogenous salicylic acid. Proc Natl Acad Sci U S A. 1989:86:2214–2218. 10.1073/pnas.86.7.2214. 16594020 PMC286882

[kiaf615-B92] Rate DN, Greenberg JT. The Arabidopsis aberrant growth and death2 mutant shows resistance to *Pseudomonas syringae* and reveals a role for NPR1 in suppressing hypersensitive cell death. Plant J. 2001:27:203–211. 10.1046/j.0960-7412.2001.1075umedoc.x. 11532166

[kiaf615-B93] Régnier CH et al Identification and characterization of an IκB kinase. Cell. 1997:90:373–383. 10.1016/S0092-8674(00)80344-X. 9244310

[kiaf615-B94] Rhoads DM, McIntosh L. Salicylic acid regulation of respiration in higher plants: alternative oxidase expression. Plant Cell. 1992:4:1131–1139. 10.2307/3869481. 12297672 PMC160203

[kiaf615-B95] Roden LC, Ingle RA. Lights, rhythms, infection: the role of light and the circadian clock in determining the outcome of plant-pathogen interactions. Plant Cell. 2009:21:2546–2552. 10.1105/tpc.109.069922. 19789275 PMC2768925

[kiaf615-B96] Ross AF . Systemic acquired resistance induced by localized virus infections in plants. Virology. 1961:14:340–358. 10.1016/0042-6822(61)90319-1. 13743578

[kiaf615-B97] Rushton PJ et al Interaction of elicitor-induced DNA-binding proteins with elicitor response elements in the promoters of parsley PR1 genes. EMBO J. 1996:15:5690–5700. 10.1002/j.1460-2075.1996.tb00953.x. 8896462 PMC452313

[kiaf615-B98] Ryals J et al The Arabidopsis NIM1 protein shows homology to the mammalian transcription factor inhibitor I kappa B. Plant Cell. 1997:9:425–439. 10.1105/tpc.9.3.425. 9090885 PMC156928

[kiaf615-B99] Ryan CS, Schroder M. The human DEAD-box helicase DDX3X as a regulator of mRNA translation. Front Cell Dev Biol. 2022:10:1033684. 10.3389/fcell.2022.1033684. 36393867 PMC9642913

[kiaf615-B100] Saleh A et al Posttranslational modifications of the master transcriptional regulator NPR1 enable dynamic but tight control of plant immune responses. Cell Host Microbe. 2015:18:169–182. 10.1016/j.chom.2015.07.005. 26269953 PMC4537515

[kiaf615-B101] Salmeron JM et al Tomato Prf is a member of the leucine-rich repeat class of plant disease resistance genes and lies embedded within the Pto kinase gene cluster. Cell. 1996:86:123–133. 10.1016/S0092-8674(00)80083-5. 8689679

[kiaf615-B102] Sarge KD, Murphy SP, Morimoto RI. Activation of heat shock gene transcription by heat shock factor 1 involves oligomerization, acquisition of DNA-binding activity, and nuclear localization and can occur in the absence of stress. Mol Cell Biol. 1993:13:1392–1407. 10.1128/mcb.13.3.1392-1407.1993. 8441385 PMC359449

[kiaf615-B103] Seluzicki A, Burko Y, Chory J. Dancing in the dark: darkness as a signal in plants. Plant Cell Environ. 2017:40:2487–2501. 10.1111/pce.12900. 28044340 PMC6110299

[kiaf615-B104] Sen ND, Zhou F, Ingolia NT, Hinnebusch AG. Genome-wide analysis of translational efficiency reveals distinct but overlapping functions of yeast DEAD-box RNA helicases Ded1 and eIF4A. Genome Res. 2015:25:1196–1205. 10.1101/gr.191601.115. 26122911 PMC4510003

[kiaf615-B105] Shah J, Tsui F, Klessig DF. Characterization of a salicylic acid-insensitive mutant (sai1) of *Arabidopsis thaliana*, identified in a selective screen utilizing the SA-inducible expression of the tms2 gene. Mol Plant Microbe Interact. 1997:10:69–78. 10.1094/MPMI.1997.10.1.69. 9002272

[kiaf615-B106] Sheard LB et al Jasmonate perception by inositol-phosphate-potentiated COI1-JAZ co-receptor. Nature. 2010:468:400–405. 10.1038/nature09430. 20927106 PMC2988090

[kiaf615-B107] Siegfried NA, Busan S, Rice GM, Nelson JA, Weeks KM. RNA motif discovery by SHAPE and mutational profiling (SHAPE-MaP). Nat Methods. 2014:11:959–965. 10.1038/nmeth.3029. 25028896 PMC4259394

[kiaf615-B108] Silva KJP, Mahna N, Mou Z, Folta KM. NPR1 as a transgenic crop protection strategy in horticultural species. Hortic Res. 2018:5:15. 10.1038/s41438-018-0026-1. 29581883 PMC5862871

[kiaf615-B109] Slusarenko AJ, Schlaich NL. Downy mildew of *Arabidopsis thaliana* caused by Hyaloperonospora parasitica (formerly *Peronospora parasitica*). Mol Plant Pathol. 2003:4:159–170. 10.1046/j.1364-3703.2003.00166.x. 20569375

[kiaf615-B110] Sonenberg N, Hinnebusch AG. Regulation of translation initiation in eukaryotes: mechanisms and biological targets. Cell. 2009:136:731–745. 10.1016/j.cell.2009.01.042. 19239892 PMC3610329

[kiaf615-B111] Spoel SH et al Proteasome-mediated turnover of the transcription coactivator NPR1 plays dual roles in regulating plant immunity. Cell. 2009:137:860–872. 10.1016/j.cell.2009.03.038. 19490895 PMC2704463

[kiaf615-B112] Sun C et al Precise integration of large DNA sequences in plant genomes using PrimeRoot editors. Nat Biotechnol. 2024:42:316–327. 10.1038/s41587-023-01769-w. 37095350

[kiaf615-B113] Tada Y et al Plant immunity requires conformational changes [corrected] of NPR1 via S-nitrosylation and thioredoxins. Science. 2008:321:952–956. 10.1126/science.1156970. 18635760 PMC3833675

[kiaf615-B114] Tai TH et al Expression of the *Bs2* pepper gene confers resistance to bacterial spot disease in tomato. Proc Natl Acad Sci U S A. 1999:96:14153–14158. 10.1073/pnas.96.24.14153. 10570214 PMC24206

[kiaf615-B115] Tan X et al Mechanism of auxin perception by the TIR1 ubiquitin ligase. Nature. 2007:446:640–645. 10.1038/nature05731. 17410169

[kiaf615-B116] Tang Y, Ho MI, Kang BH, Gu Y. GBPL3 localizes to the nuclear pore complex and functionally connects the nuclear basket with the nucleoskeleton in plants. PLoS Biol. 2022:20:e3001831. 10.1371/journal.pbio.3001831. 36269771 PMC9629626

[kiaf615-B117] Torres MA, Jones JD, Dangl JL. Reactive oxygen species signaling in response to pathogens. Plant Physiol. 2006:141:373–378. 10.1104/pp.106.079467. 16760490 PMC1475467

[kiaf615-B118] Ugalde JM et al Chloroplast-derived photo-oxidative stress causes changes in H_2_O_2_ and EGSH in other subcellular compartments. Plant Physiol. 2021:186:125–141. 10.1093/plphys/kiaa095. 33793922 PMC8154069

[kiaf615-B119] Uknes S et al Acquired resistance in Arabidopsis. Plant Cell. 1992:4:645–656. 10.1105/tpc.4.6.645. 1392589 PMC160161

[kiaf615-B120] van den Heuvel S . Protein degradation: CUL-3 and BTB–partners in proteolysis. Curr Biol. 2004:14:R59–R61. 10.1016/j.cub.2003.12.044. 14738749

[kiaf615-B121] Van der Biezen EA, Jones JD. Plant disease-resistance proteins and the gene-for-gene concept. Trends Biochem Sci. 1998:23:454–456. 10.1016/S0968-0004(98)01311-5. 9868361

[kiaf615-B122] van der Hoorn RA, Kamoun S. From guard to decoy: a new model for perception of plant pathogen effectors. Plant Cell. 2008:20:2009–2017. 10.1105/tpc.108.060194. 18723576 PMC2553620

[kiaf615-B123] Vishwakarma A, Tetali SD, Selinski J, Scheibe R, Padmasree K. Importance of the alternative oxidase (AOX) pathway in regulating cellular redox and ROS homeostasis to optimize photosynthesis during restriction of the cytochrome oxidase pathway in *Arabidopsis thaliana*. Ann Bot. 2015:116:555–569. 10.1093/aob/mcv122. 26292995 PMC4578005

[kiaf615-B124] Wang D, Amornsiripanitch N, Dong X. A genomic approach to identify regulatory nodes in the transcriptional network of systemic acquired resistance in plants. PLoS Pathog. 2006:2:e123. 10.1371/journal.ppat.0020123. 17096590 PMC1635530

[kiaf615-B125] Wang D, Weaver ND, Kesarwani M, Dong X. Induction of protein secretory pathway is required for systemic acquired resistance. Science. 2005:308:1036–1040. 10.1126/science.1108791. 15890886

[kiaf615-B126] Wang J, Zhang X, Greene GH, Xu G, Dong X. PABP/purine-rich motif as an initiation module for cap-independent translation in pattern-triggered immunity. Cell. 2022:185:3186–3200 e3117. 10.1016/j.cell.2022.06.037. 35907403 PMC9391319

[kiaf615-B127] Wang S et al The role of TIR domain-containing proteins in bacterial defense against phages. Nat Commun. 2024:15:7384. 10.1038/s41467-024-51738-3. 39191765 PMC11350192

[kiaf615-B128] Wang W et al Timing of plant immune responses by a central circadian regulator. Nature. 2011:470:110–114. 10.1038/nature09766. 21293378 PMC6601609

[kiaf615-B129] Wang W et al Structural basis of salicylic acid perception by Arabidopsis NPR proteins. Nature. 2020:586:311–316. 10.1038/s41586-020-2596-y. 32788727 PMC7554156

[kiaf615-B130] Wang Y et al Deciphering phenylalanine-derived salicylic acid biosynthesis in plants. Nature. 2025:645:208–217. 10.1038/s41586-025-09280-9. 40702180 PMC12408371

[kiaf615-B131] Ward ER et al Coordinate gene activity in response to agents that induce systemic acquired resistance. Plant Cell. 1991:3:1085–1094. 10.2307/3869297. 12324583 PMC160074

[kiaf615-B132] Whalen MC, Innes RW, Bent AF, Staskawicz BJ. Identification of *Pseudomonas syringae* pathogens of Arabidopsis and a bacterial locus determining avirulence on both Arabidopsis and soybean. Plant Cell. 1991:3:49–59. 10.1105/tpc.3.1.49. 1824334 PMC159978

[kiaf615-B133] White RF . Acetylsalicylic acid (aspirin) induces resistance to tobacco mosaic virus in tobacco. Virology. 1979:99:410–412. 10.1016/0042-6822(79)90019-9. 18631626

[kiaf615-B134] Whitham S et al The product of the tobacco mosaic virus resistance gene N: similarity to toll and the interleukin-1 receptor. Cell. 1994:78:1101–1115. 10.1016/0092-8674(94)90283-6. 7923359

[kiaf615-B135] Womble DD, Dong X, Luckow VA, Wu RP, Rownd RH. Analysis of the individual regulatory components of the IncFII plasmid replication control system. J Bacteriol. 1985:161:534–543. 10.1128/jb.161.2.534-543.1985. 3155721 PMC214915

[kiaf615-B136] Woronicz JD, Gao X, Cao Z, Rothe M, Goeddel DV. Iκb kinase-beta: NF-κB activation and complex formation with IκB kinase-alpha and NIK. Science. 1997:278:866–869. 10.1126/science.278.5339.866. 9346485

[kiaf615-B137] Xiang Y et al Pervasive downstream RNA hairpins dynamically dictate start-codon selection. Nature. 2023:621:423–430. 10.1038/s41586-023-06500-y. 37674078 PMC10499604

[kiaf615-B138] Xiang Y, Dong X. Translational regulation of plant stress responses: mechanisms, pathways, and applications in bioengineering. Annu Rev Phytopathol. 2025:63:117–146. 10.1146/annurev-phyto-121823-032335. 40455831 PMC12515504

[kiaf615-B139] Xin XF et al Bacteria establish an aqueous living space in plants crucial for virulence. Nature. 2016:539:524–529. 10.1038/nature20166. 27882964 PMC5135018

[kiaf615-B140] Xu G et al Global translational reprogramming is a fundamental layer of immune regulation in plants. Nature. 2017a:545:487–490. 10.1038/nature22371. 28514447 PMC5485861

[kiaf615-B141] Xu G et al uORF-mediated translation allows engineered plant disease resistance without fitness costs. Nature. 2017b:545:491–494. 10.1038/nature22372. 28514448 PMC5532539

[kiaf615-B142] Xu G, Moeder W, Yoshioka K, Shan L. A tale of many families: calcium channels in plant immunity. Plant Cell. 2022:34:1551–1567. 10.1093/plcell/koac033. 35134212 PMC9048905

[kiaf615-B143] Yoo H et al Translational regulation of metabolic dynamics during effector-triggered immunity. Mol Plant. 2020:13:88–98. 10.1016/j.molp.2019.09.009. 31568832 PMC6946852

[kiaf615-B144] Yu C, Huang L. New advances in cross-linking mass spectrometry toward structural systems biology. Curr Opin Chem Biol. 2023:76:102357. 10.1016/j.cbpa.2023.102357. 37406423 PMC11091472

[kiaf615-B145] Yuan M, Ngou BPM, Ding P, Xin XF. PTI-ETI crosstalk: an integrative view of plant immunity. Curr Opin Plant Biol. 2021:62:102030. 10.1016/j.pbi.2021.102030. 33684883

[kiaf615-B146] Zandi E, Rothwarf DM, Delhase M, Hayakawa M, Karin M. The IκB kinase complex (IKK) contains two kinase subunits, IKKα and IKKβ, necessary for IκB phosphorylation and NF-κB activation. Cell. 1997:91:243–252. 10.1016/S0092-8674(00)80406-7. 9346241

[kiaf615-B147] Zavaliev R, Dong X. NPR1, a key immune regulator for plant survival under biotic and abiotic stresses. Mol Cell. 2024:84:131–141. 10.1016/j.molcel.2023.11.018. 38103555 PMC10929286

[kiaf615-B148] Zavaliev R, Mohan R, Chen T, Dong X. Formation of NPR1 condensates promotes cell survival during the plant immune response. Cell. 2020:182:1093–1108.e18. 10.1016/j.cell.2020.07.016. 32810437 PMC7484032

[kiaf615-B149] Zhang H et al Determinants of genome-wide distribution and evolution of uORFs in eukaryotes. Nat Commun. 2021:12:1076. 10.1038/s41467-021-21394-y. 33597535 PMC7889888

[kiaf615-B150] Zhang Q, Lenardo MJ, Baltimore D. 30 years of NF-κB: a blossoming of relevance to human pathobiology. Cell. 2017:168:37–57. 10.1016/j.cell.2016.12.012. 28086098 PMC5268070

[kiaf615-B151] Zhang Y, Fan W, Kinkema M, Li X, Dong X. Interaction of NPR1 with basic leucine zipper protein transcription factors that bind sequences required for salicylic acid induction of the *PR-1* gene. Proc Natl Acad Sci U S A. 1999:96:6523–6528. 10.1073/pnas.96.11.6523. 10339621 PMC26915

[kiaf615-B152] Zhang Y, Goritschnig S, Dong X, Li X. A gain-of-function mutation in a plant disease resistance gene leads to constitutive activation of downstream signal transduction pathways in suppressor of npr1-1, constitutive 1. Plant Cell. 2003:15:2636–2646. 10.1105/tpc.015842. 14576290 PMC280567

[kiaf615-B153] Zheng XY et al Spatial and temporal regulation of biosynthesis of the plant immune signal salicylic acid. Proc Natl Acad Sci U S A. 2015:112:9166–9173. 10.1073/pnas.1511182112. 26139525 PMC4522758

[kiaf615-B154] Zhou JM et al NPR1 differentially interacts with members of the TGA/OBF family of transcription factors that bind an element of the PR-1 gene required for induction by salicylic acid. Mol Plant Microbe Interact. 2000:13:191–202. 10.1094/MPMI.2000.13.2.191. 10659709

[kiaf615-B155] Zhou JM, Chai J. Plant pathogenic bacterial type III effectors subdue host responses. Curr Opin Microbiol. 2008:11:179–185. 10.1016/j.mib.2008.02.004. 18372208

[kiaf615-B156] Zhou M et al Redox rhythm reinforces the circadian clock to gate immune response. Nature. 2015:523:472–476. 10.1038/nature14449. 26098366 PMC4526266

[kiaf615-B157] Zhu B et al Complete biosynthesis of salicylic acid from phenylalanine in plants. Nature. 2025:645:218–227. 10.1038/s41586-025-09175-9. 40702181 PMC12408352

[kiaf615-B158] Zhu Y, Qian W, Hua J. Temperature modulates plant defense responses through NB-LRR proteins. PLoS Pathog. 2010:6:e1000844. 10.1371/journal.ppat.1000844. 20368979 PMC2848567

[kiaf615-B159] Zipfel C et al Bacterial disease resistance in Arabidopsis through flagellin perception. Nature. 2004:428:764–767. 10.1038/nature02485. 15085136

[kiaf615-B160] Zipfel C, Rathjen JP. Plant immunity: AvrPto targets the frontline. Curr Biol. 2008:18:R218–R220. 10.1016/j.cub.2008.01.016. 18334200

